# Increased Risk of Acute Coronary Syndrome in Patients With Chronic Pancreatitis: A Nationwide Cohort Analysis

**DOI:** 10.1097/MD.0000000000003451

**Published:** 2016-05-20

**Authors:** Ming-Tse Hsu, Cheng-Li Lin, Wei-Sheng Chung

**Affiliations:** From the Division of Gastroenterology (M-TH), Department of Internal Medicine, Chia-Yi Christian Hospital, Chiayi; Management Office for Health Data (C-LL), China Medical University Hospital; College of Medicine (C-LL), China Medical University; Department of Internal Medicine (W-SC), Taichung Hospital, Ministry of Health and Welfare; Department of Health Services Administration (W-SC), China Medical University; and Department of Healthcare Administration (W-SC), Central Taiwan University of Science and Technology, Taichung, Taiwan.

## Abstract

Chronic inflammation may promote development of coronary heart disease. Studies on the relationship between chronic pancreatitis (CP) and cardiovascular diseases are scant.

We conducted a nationwide retrospective cohort study to determine the risk of acute coronary syndrome (ACS) in patients with CP.

We randomly selected a comparison cohort of individuals without CP from the Taiwan National Health Insurance Research Database (N = 23.74 million) and frequency-matched them with patients with CP from 2000 to 2010 in a 1:4 ratio according to age, sex, and index year. The follow-up period lasted from the index date of the new CP diagnosis to the date of ACS diagnosis, censoring, or the end of 2011. We analyzed the risk of ACS by using Cox proportional-hazard models.

In total, 17,405 patients with CP and 69,620 individuals without CP were followed for 84,430 and 417,426 person-years. Most patients with CP were men, and the mean age of the patients was 48.3 ± 15.0 years. The overall ACS incidence was 2.15-fold higher in the CP cohort than in the non-CP cohort (4.89 vs 2.28 per 10,000 person-years) with an adjusted hazard ratio (aHR) of 1.40 (95% confidence interval [CI] 1.20–1.64). Compared with individuals without CP, patients with CP aged ≤39 years exhibited the highest risk of ACS (aHR 2.14, 95% CI 1.13–4.02), followed by those aged 40 to 54 years (aHR 1.66, 95% CI 1.23–2.24) and those aged 55 to 69 years (aHR 1.53, 95% CI 1.15–2.03).

CP may become an independent risk factor for ACS.

## INTRODUCTION

Chronic pancreatitis (CP) is defined as chronic inflammation and fibrosis of the pancreas, resulting in irreversible morphological changes and functional abnormalities.^1^ The worldwide increase in the prevalence of CP is attributable to the increase in alcohol consumption and the increased availability of high-quality diagnostic modalities.^2–5^ Patients with CP may experience unremitting abdominal pain, chronic diarrhea, maldigestion, glucose intolerance, and weight loss, all of which substantially impair their quality of life.^6^ Moreover, CP requires repeated medical interventions and hospitalization, and increases the burden on medical resources.^7–9^

Heavy drinking increases the risk of high blood pressure, heart failure, and stroke.^10–12^ Alcohol abuse is a prominent cause of CP.^2,13^ Evidence reveals that mild-to-moderate alcohol consumption exerts a protective effect against coronary heart disease.^14,15^ However, chronic inflammation in CP can activate immune cells to promote atherosclerotic lesions, subsequently leading to acute coronary syndrome (ACS).^16^ Unstable angina and myocardial infarction constitute ACS, causing a sudden decrease in blood flow in the coronary arteries. ACS can cause ventricular arrhythmia, cardiovascular collapse, and death despite advanced treatment options. Although hypertension, diabetes, and hyperlipidemia are well-established risk factors for ACS, approximately half of the patients with ACS do not exhibit these risk factors.^17^ Most studies on the CP focused on treatment and the risk of pancreatic neoplasm.^18–20^ Data on patients with CP and ACS prevalence are scant. Therefore, we conducted a nationwide longitudinal cohort study to evaluate the incidence and risk of ACS in patients with CP.

## METHODS

### Data Source

In March 1995, the Ministry of Health and Welfare in Taiwan integrated 13 health insurance agencies into a nationwide, universal National Health Insurance (NHI) program. The NHI program covers over 99% of the 23.74 million residents of Taiwan (http://www.nhi.gov.tw). The National Health Research Institutes (NHRI) maintains the claims data of beneficiaries enrolled in the NHI program. The NHRI has established and released the National Health Insurance Research Database (NHIRD) annually to the public for research; all data related to personal identification are encrypted by the National Health Insurance Administration (NHIA) before release. In this study, we used a subset of the NHIRD containing healthcare data, such as inpatient claims and the registry of beneficiaries. All clinical diagnoses were recorded using the “International Classification of Diseases, Ninth Revision, Clinical Modification” (ICD-9-CM) codes.^21^ The study was exempted from a full review by the institutional research ethic committee (CMUH-104-REC2–115). The reliability and validity of this NHIRD database have been published.^22,23^

### Study Design

The study design is a nationwide retrospective cohort study.

### Sampled Participants

From the inpatient claims, we selected all adult patients with a first-time diagnosis of CP (ICD-9-CM 577.1) between 2000 and 2010 as the CP cohort. The date of CP diagnosis were defined as the index date. The recurrence rate of ACS remains high.^24^ Pancreatic cancer has a low survival rate in 1 year.^25^ Therefore, we excluded those with a history of ACS (ICD-9-CM 410, 411.1, and 411.8) or pancreatic cancer (ICD-9-CM 157) at the baseline. We also excluded patients aged <20 years, those with incomplete age or sex information. A non-CP comparison cohort was randomly selected from the NHI comprising beneficiaries aged ≥20 years and frequency-matched with the CP cohort in a 4:1 ratio according to age (every 5 years), sex, and the year of index date, with the same exclusion criteria as that of the CP cohort.

### Exposure Variables

In Taiwan, the diagnosis of CP is made by physicians based on the clinical presentation and imaging studies, namely contrast-enhanced computer tomography, ultrasonography, magnetic resonance image, or endoscopic retrograde cholangiopancreatography.

### Outcome and Comorbidities

The outcome of interest was new ACS diagnosis between 2000 and 2011. The baseline comorbidities were hypertension (ICD-9-CM 401–405), diabetes (ICD-9-CM 250), hyperlipidemia (ICD-9-CM 272), cerebrovascular accident (CVA; ICD-9-CM 430–438), atrial fibrillation (ICD-9-CM 427.31), heart failure (ICD-9-CM 428), chronic obstructive pulmonary disease (COPD; ICD-9-CM 491, 492, and 496), chronic kidney disease (CKD; ICD-9-CM 580–589), and acute pancreatitis (ICD-9-CM 577.0), all of which were identified from their diagnoses in the hospitalization records before the index date.

### Statistical Analysis

Demographic characteristics and the prevalence of comorbidities in the CP and non-CP cohorts were compared using a chi-square test for categorical variables and a *t* test for continuous variables. The incidence densities of ACS were calculated according to sex, age, and the presence of comorbidities for each cohort. Univariable and multivariable Cox proportion-hazard regression models were used to examine the effect of CP on the risk of ACS and were expressed using hazard ratios (HRs) with a 95% confidence interval (CI). The multivariable model was simultaneously adjusted for age, sex, and comorbidities of hypertension, diabetes, hyperlipidemia, CVA, atrial fibrillation, heart failure, COPD, CKD, and acute pancreatitis. Additional data analysis was performed to evaluate the joint effect between CP and the ACS-associated risk factors on ACS. The Kaplan–Meier method was used for estimating the cumulative incidence of ACS between the CP and non-CP cohorts, and the differences were assessed using a log-rank test. All statistical analyses were performed using SAS version 9.3 (SAS Institute, Inc., Cary, NC). *P* < 0.05 was considered significant.

## RESULTS

**FIGURE 1:**
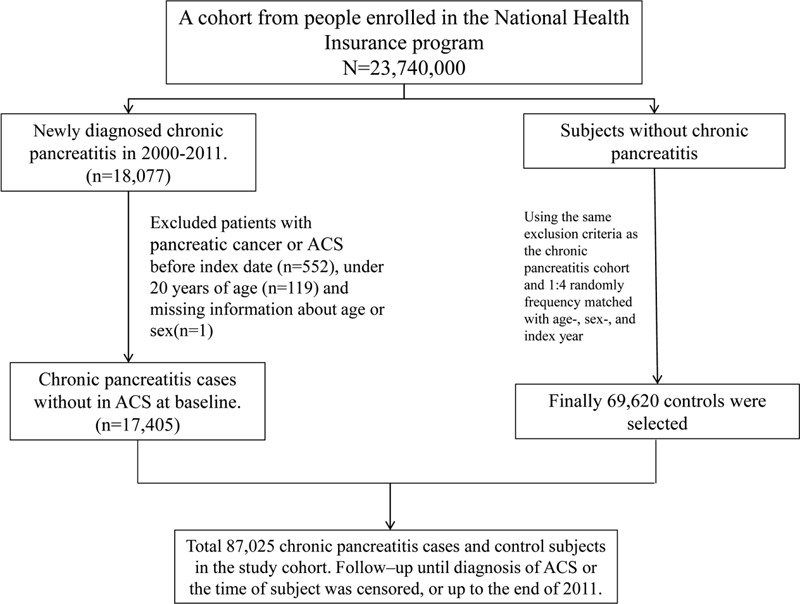
A flow diagram of the study participants.

The average annual incidence rate of CP between 2000 and 2010 were 9.50 per 100,000 persons (data not shown). A flow diagram of the study participants is depicted in Figure 1. We identified 17,405 patients with CP and matched them with 69,620 participants without CP. The baseline characteristics of patients in the 2 cohorts are presented in Table 1. Approximately 73.5% of patients were aged <54 years, and most patients were men (82.8% vs 17.2%). The mean ages of the participants were 48.3 ± 15.0 and 47.9 ± 15.3 years in the CP and non-CP cohorts, respectively. Patients with CP had a higher prevalence of comorbidities than those without CP (all *P* < 0.001).

**TABLE 1:**
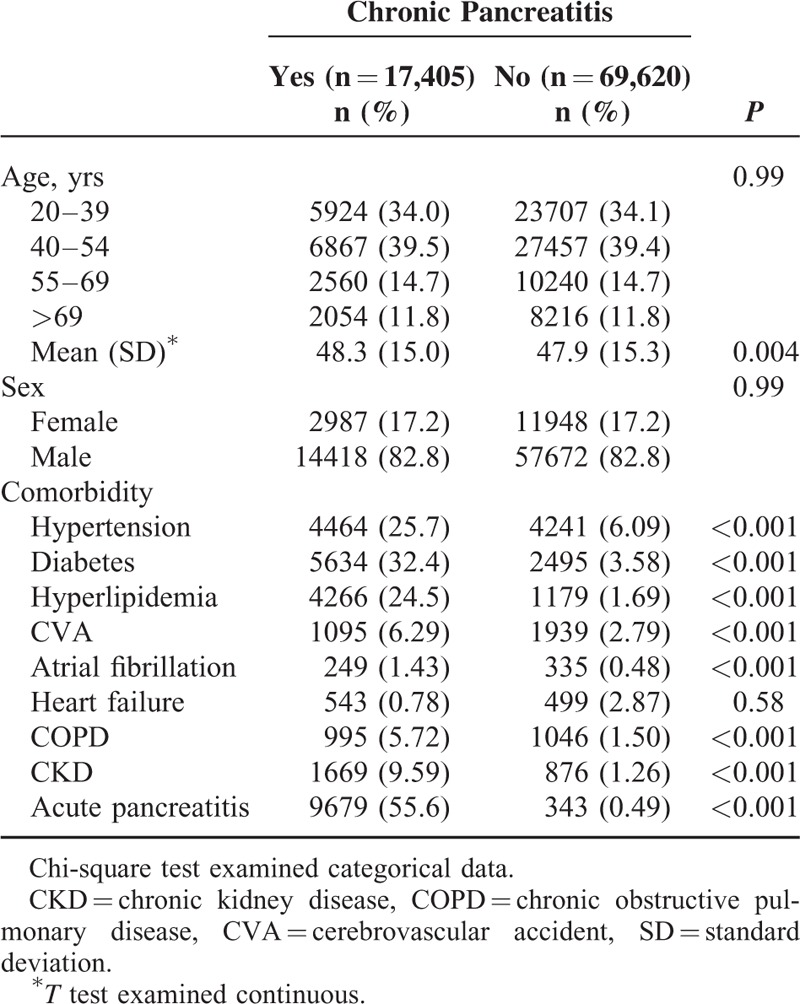
Comparison of Demographics and Comorbidity Between Chronic Pancreatitis Patients and Controls

**FIGURE 2:**
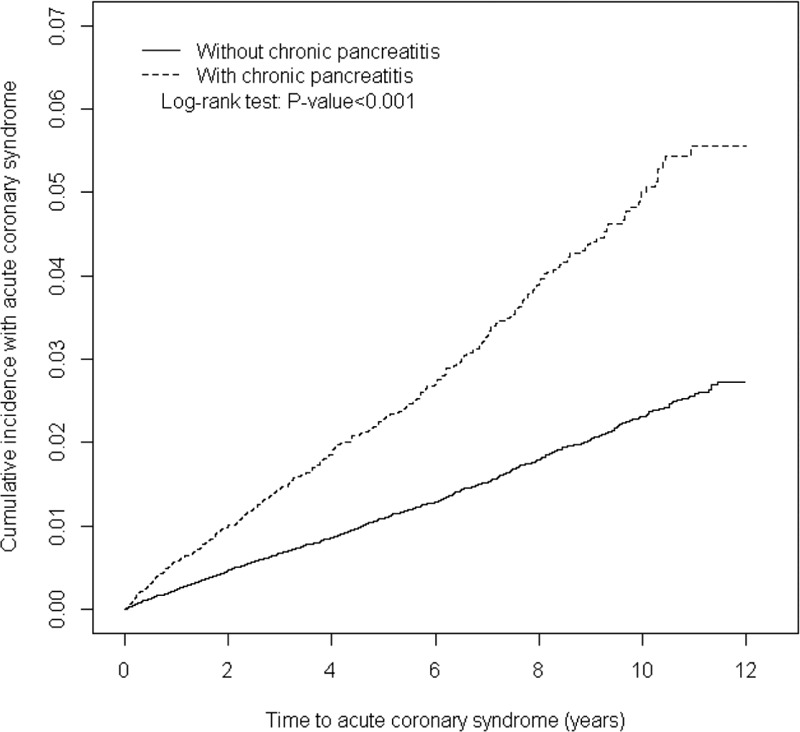
Cummulative incidence of ACS in patients with chronic pancreatitis and comparison patients. ACS = acute coronary syndrome.

During the mean follow-up period of 4.85 and 6.00 years for the CP and non-CP cohorts, respectively, the cumulative incidence of ACS was significantly higher for patients in the CP cohort than in the non-CP cohort (log-rank test *P* < 0.001; Figure 2). The overall incidence of ACS was 2.15-fold higher in the CP cohort than in the non-CP cohort (4.89 vs 2.28 per 10,000 person-years), with an adjusted HR (aHR) of 1.40 (95% CI 1.20–1.64; Table 2). The incidence of ACS was 6.33 and 4.60 per 10,000 person-years for men and women, respectively, in the CP cohort. The sex-specific relative risk of ACS was significantly higher for men in the CP cohort than in the non-CP cohort (aHR 1.44, 95% CI 1.21–1.72). Compared with individuals without CP, patients with CP aged ≤39 years exhibited the highest risk of ACS (aHR 2.14, 95% CI 1.13–4.02), followed by those aged 40 to 54 years (aHR 1.66, 95% CI 1.23–2.24) and those aged 55 to 69 years (aHR 1.53, 95% CI 1.15–2.03). The incidence of ACS was higher in patients with comorbidities in both cohorts. In patients without comorbidities, the risk of ACS was consistently higher in the CP cohort than in the non-CP cohort (aHR 1.41, 95% CI 1.05–1.92).

**TABLE 2:**
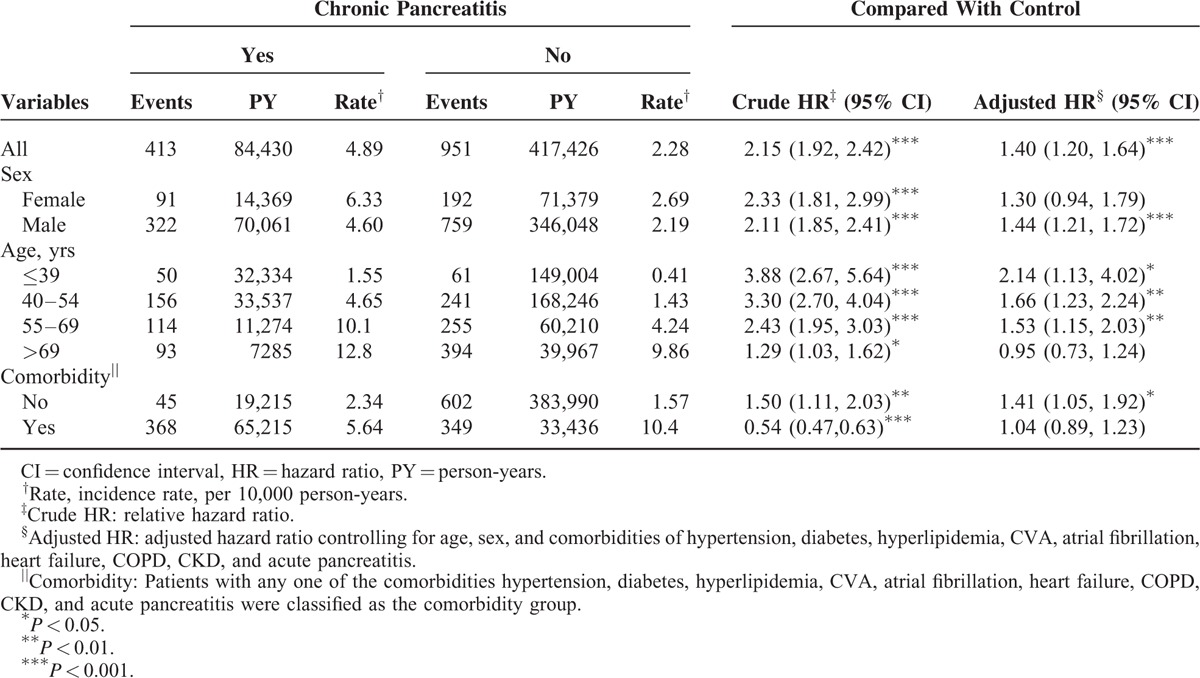
Comparison of Incidence and Adjusted Hazard Ratio of ACS by Sex, Age and Comorbidity Between Chronic Pancreatitis Patients and Controls

Compared with individuals without CP and lacking comorbidities, those with only diabetes (aHR 3.31, 95% CI 2.42–4.54) and those with only hypertension (aHR 2.12, 95% CI 1.62–2.77) had a higher risk of ACS. Moreover, compared with individuals without CP lacking comorbidities, patients with CP with 5 or more comorbidities were at a significantly higher risk of ACS (aHR 9.67, 95% CI 7.08–13.2), followed by those with 4 comorbidities (aHR 5.82, 95% CI 4.37–7.74), those with 3 comorbidities (aHR 4.47, 95% CI 3.57–5.61), those with 2 comorbidities (aHR 3.07, 95% CI 2.48–3.80), and those with 1 comorbidity (aHR 2.14, 95% CI 1.71–2.68; Table 3).

**TABLE 3:**
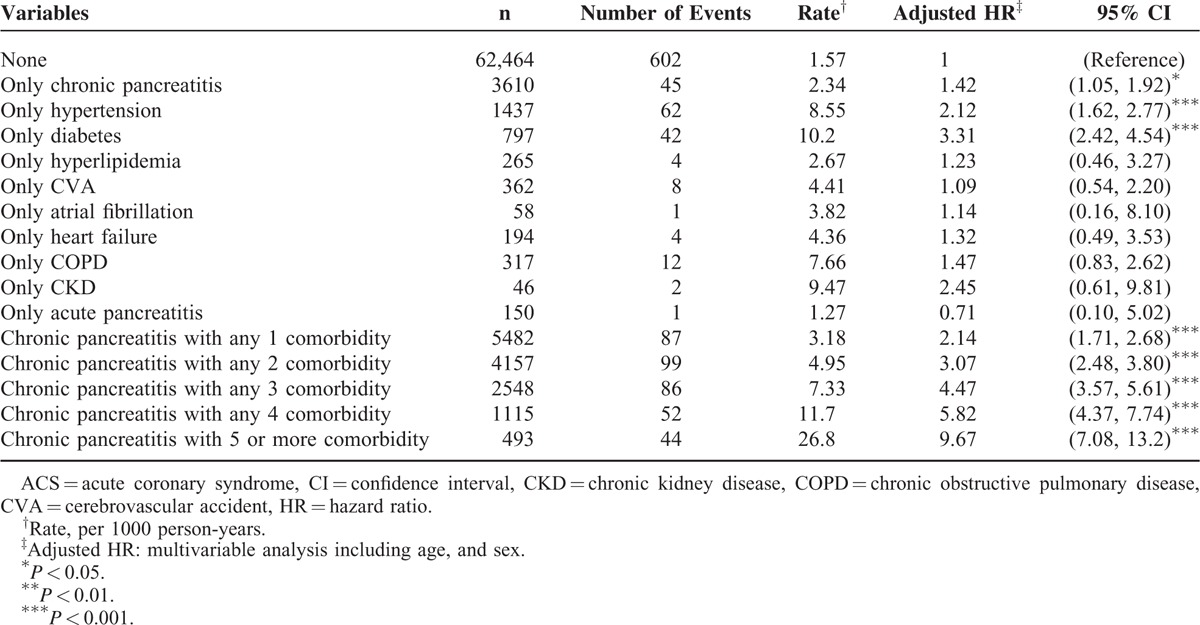
Joint Effects for ACS Between Chronic Pancreatitis and ACS-associated Risk Factors

## DISCUSSION

The current study demonstrated that the overall incidence of ACS was 2.15-fold higher in the CP cohort than in the comparison cohort (4.89 vs 2.28 per 10,000 person-years). Patients in the CP cohort exhibited a higher proportion of comorbidities, except heart failure, than those in the comparison cohort did. After adjustment for covariates, patients with CP were at a 1.40-fold increased risk of ACS (aHR 1.40, 95% CI 1.20–1.64) compared with those without CP.

Endothelium is considered a regulator for blood flow and vascular homeostasis. Inflammation may cause endothelial dysfunction, which contributes to the initiation and progression of atherosclerotic diseases.^26–28^ Infiltration of macrophages and T lymphocytes in the pancreas has been established in CP.^29–31^ These inflammatory cells once enhance inflammation and tend to precipitate unstable or ruptured atherosclerotic plaques in the coronary arteries.^32^ In addition, the macrophages may produce prothrombotic and procoagulant factors enhancing thrombus formation at the site of plaque rupture.^16^

The activated pancreatic stellate cells (PSCs) induce fibrosis in CP.^33^ Several inflammatory cytokines, namely tumor necrosis factor-alpha (TNF-α), interleukin (IL)-1, and IL-6, can activate the PSCs. IL-6 presents at the atherosclerotic plaques and may enhance plaque instability.^34^ Secretion of TNF-α, a proinflammatory cytokine, is high in patients with acute myocardial ischemia and recurrent myocardial infarction, and may result in cardiac death.^35^

Most patients with CP in the current study were men, and their mean age was 48.3 ± 15.0 years. The findings of this study are consistent with those of previous reports.^36^ Although the incidence of ACS was higher in patients with CP than in those without CP for both sexes, men with CP were at a significantly higher risk of ACS than those without CP (aHR 1.44, 95% CI 1.21–1.72). Patients with CP aged ≤69 years were at a significantly increased risk of subsequent ACS compared with their counterparts without CP. Studies have reported that proinflammatory cytokines and coagulation factor production increase with age and cause frailty.^37,38^ Moreover, the prevalence of comorbidities is high in older adults, which may dilute the effect of CP.^39^ Therefore, clinicians should proactively examine men and adults aged ≤69 years with CP to prevent ACS.

Our study has several strengths. We determined that patients with CP are at an increased risk of subsequent ACS by using a nationwide epidemiologic design. We enrolled a large sample. Moreover, the NHI is mandatory in Taiwan, and the NHI beneficiaries are assigned unique personal identification numbers, enabling researchers to trace them throughout the follow-up period. Thus, our findings can be generalized to the entire population of Taiwan.

Several limitations must be considered while interpreting these findings. First, the NHIRD does not provide detailed personal information, such as smoking habits, body mass index, and physical activity levels, all of which are potential confounding factors in this study. Hypertension, COPD, and CVA are well-established comorbidities related to tobacco smoking and obesity.^40–43^ We adjusted the model for hypertension, COPD, and CVA to mediate the influence of smoking and obesity. Second, the lack of drug-treatment data, for example, those on statin therapy and using anticoagulants and antiplatelet drugs, is another limitation of this study. Finally, the potential misclassification bias of interest outcomes may exist in the healthcare claims data; however, the auditing mechanism of the NHIA can facilitate minimizing diagnostic uncertainty and misclassification. Although our meticulous study design adjusts for confounders, a key limitation of this study lies the potential for bias caused by possible unmeasured or unknown confounders.

In conclusion, this nationwide cohort study of 17,405 patients with CP, with 85,000 person-years of follow-up, demonstrated that patients with CP are at a 1.40-fold increased risk of ACS compared with the general population. Men with CP were at a 1.44-fold higher risk of ACS than those without CP. Patients with CP aged ≤69 years were at a significantly increased risk of subsequent ACS compared with their counterparts without CP. Clinicians should be aware of ACS risks in patients with CP and provide appropriate cardiovascular care in addition to CP treatment. However, future prospective studies are warranted to affirm the findings because of the retrospective observational design employed in this study.
